# The Immune Cell Landscape in Different Anatomical Structures of Knee in Osteoarthritis: A Gene Expression-Based Study

**DOI:** 10.1155/2020/9647072

**Published:** 2020-03-19

**Authors:** Ziming Chen, Yuanchen Ma, Xuerui Li, Zhantao Deng, Minghao Zheng, Qiujian Zheng

**Affiliations:** ^1^Centre for Orthopaedic Translational Research, Medical School, University of Western Australia, Nedlands, Australia; ^2^Department of Orthopedics, Guangdong Provincial People's Hospital (Guangdong Academy of Medical Sciences), Guangdong Province, China; ^3^Goldsmiths, University of London, London, UK

## Abstract

**Background:**

Immunological mechanisms play a vital role in the pathogenesis of knee osteoarthritis (KOA). Moreover, the immune phenotype is a relevant prognostic factor in various immune-related diseases. In this study, we used CIBERSORT for deconvolution of global gene expression data to define the immune cell landscape of different structures of knee in osteoarthritis. *Methods and Findings*. By applying CIBERSORT, we assessed the relative proportions of immune cells in 76 samples of knee cartilage, 146 samples of knee synovial tissue, 40 samples of meniscus, and 50 samples of knee subchondral bone. Enumeration and activation status of 22 immune cell subtypes were provided by the obtained immune cell profiles. In synovial tissues, the differences in proportions of plasma cells, M1 macrophages, M2 macrophages, activated dendritic cells, resting mast cells, and eosinophils between normal tissues and osteoarthritic tissues were statistically significant (*P* < 0.05). The area under the curve was relatively large in resting mast cells, dendritic cells, and M2 macrophages in receiver operating characteristic analyses. In subchondral bones, the differences in proportions of resting master cells and neutrophils between normal tissues and osteoarthritic tissues were statistically significant (*P* < 0.05). In subchondral bones, the proportions of immune cells, from the principle component analyses, displayed distinct group-bias clustering. Resting mast cells and T cell CD8 were the major component of first component. Moreover, we revealed the potential interaction between immune cells. There was almost no infiltration of immune cells in the meniscus and cartilage of the knee joint.

**Conclusions:**

The immune cell composition in KOA differed substantially from that of healthy joint tissue, while it also differed in different anatomical structures of the knee. Meanwhile, activated mast cells were mainly associated with high immune cell infiltration in OA. Furthermore, we speculate M2 macrophages in synovium and mast cells in subchondral bone may play an important role in the pathogenesis of OA.

## 1. Introduction

Knee osteoarthritis (KOA) is one of the most frequently common diseases in orthopedic department, which affects 30%-50% of people over 65 years old [1]. Although a series of treatment, such as anti-inflammatory medicine, play a certain role in relieving symptoms, it is difficult to prevent the process of bone degeneration, and total knee arthroplasty is still the mainly curative therapy for KOA [2].

KOA is a chronic degenerative disease characterized by articular cartilage injury and degeneration, together with sclerosis, proliferation and cystic degeneration of subchondral bone, and subsequent narrowing of articular space [3]. In osteoarthritis (OA), various anatomical structures of the knee joint are damaged. OA was used to be considered as “mechanical wear and tear” [4]. However, in recent years, more and more studies have shown that immunological mechanisms play the vital role in the pathogenesis of OA [5, 6], and OA is gradually considered as a chronic inflammatory response [7]. In the pathological process of OA, the destruction of bone and cartilage caused by synovitis and inflammation is the hot spots for series studies [8]. Until now, however, the role of various immune cells in osteoarthritis-related microenvironment still has not been clarified.

The function and proportions of infiltrating immune cells vary subtly according to the host's immune status, which is reported to be effective drug targeting and related to clinical outcomes [9–11]. Moreover, the immune phenotype is a relevant prognostic factor in various immune-related diseases [12–14]. Therefore, clarification of local infiltration of immune cells in knee joint contributes to better understand the local immune situation and to develop new treatment methods.

Immune cell composition of solid tissues is usually analyzed by flow cytometry and immunohistochemistry, which have the limitations in small number of detected cells and great need of number of fluorescence channels. The system biology tool Cell-type Identification By Estimating Relative Subsets Of known RNA Transcripts (CIBERSORT) can employ deconvolution of bulk gene expression data from solid tissues, enumerate 22 immune cell types at once, and apply signatures from ~500 marker genes to quantify the relative fraction of each cell type, which means there is a high resolving power for CIBERSORT [12, 15].

Therefore, in the present study, we used CIBERSORT for deconvolution of global gene expression data to define the immune cell landscape of different structures of knee in osteoarthritis.

## 2. Methods

### 2.1. Data Acquisition

**Figure 1:**
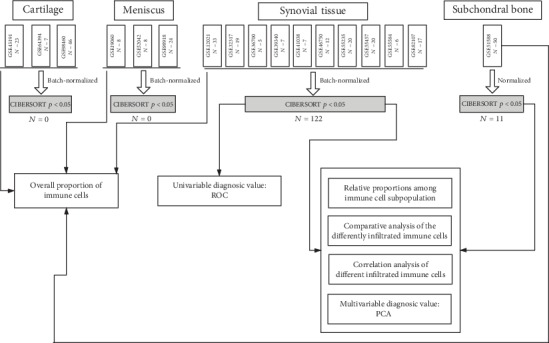
Flowchart detailing the study design. GEO: Gene Expression Omnibus; CIBERSORT: Cell-type Identification By Estimating Relative Subsets Of known RNA Transcripts; PCA: Principle component analyses; ROC: receiver operating characteristic.

In the present study, datasets were searched from the Gene Expression Omnibus (GEO) database [16] with the keywords “osteoarthritis” [MeSH Terms] OR “osteoarthritis” [All Fields] AND “Homo sapiens” [porgn] AND “gse” [Filter], uploaded up to 15 September 2019. The study type was described as “expression profiling by array.” All selected datasets were genome-wide expression data in different structures of knee of normal or OA patients. Datasets with samples of normal area in OA patients were excluded, considering it is hard to define it as normal or OA tissues. All of the selected studies were approved by their respective institutional review boards. Preprocessing, aggregation, and normalization of raw data were performed according to the robust multiarray average algorithm. Details of the study design are illustrated in Figure 1 as a flowchart.

### 2.2. Evaluation of Infiltrating Immune Cells in Different Structures of Knee

Normalized gene expression data were used to infer the relative proportions of 22 types of infiltrating immune cells using the CIBERSORT algorithm. Briefly, gene expression datasets were prepared using standard annotation files and data uploaded to the CIBERSORT web portal, with the algorithm run using the default signature matrix at 1000 permutations. CIBERSORT is an analytical tool which accurately quantifies the relative levels of distinct immune cell types within a complex gene expression mixture [15]. CIBERSORT derives a *P* value for the deconvolution for each sample using the Monte Carlo sampling, providing a measure of confidence in the results. A set of barcode gene expression values (a “signature matrix” of 547 genes) was used by CIBERSORT for characterizing immune cell composition. Here, the original CIBERSORT gene signature file LM22 was applied. The 22 cell types inferred by CIBERSORT include B cells, T cells, natural killer cells, macrophages, dendritic cells, eosinophils, and neutrophils, amongst others.

### 2.3. Principle Component Analyses (PCA) and Receiver Operating Characteristic (ROC) Analyses

Principle components analysis (PCA) was used to identify major sources of variance in the proportion of different types of infiltrating immune cells between normal patients and OA patients. The major sources of variance could potentially be the diagnostic clues for OA. When the most major variation in PCA result was still low (<30%), receiver operating characteristic (ROC) was performed to assess the diagnostic value of different types of infiltrating immune cells separately. The area under the curve (AUC) under 95% confidence interval was calculated, and the ROC curve was generated.

### 2.4. Overall Proportion of Immune Cells in Different Tissues

As explained by the creators of CIBERSORT, the CIBERSORT *P* value is empirical and produced for the deconvolution [15]. It is calculated for the actual observed data instead of theoretical data. Moreover, it tests the null hypothesis that none of the cells that comprise the signature matrix in a given sample are present. Thus, it was considered as a parameter that could reflect the proportion of a sample that comprised immune cells versus nonimmune cells, where a greater proportion of nonimmune cells would produce a correspondingly larger *P* value. Also, several researches have confirmed this hypothesis [12, 17, 18]. In the present study, high infiltration of immune cells was defined as CIBERSORT *P* value ≤ 0.01, medium infiltration of immune cells was defined as 0.01 < CIBERSORT*P* value ≤ 0.05, and low infiltration of immune cells was defined as CIBERSORT *P* value > 0.05.

### 2.5. Comparison of the Results Calculated by xCell and CIBERSORT Algorithms

In order to validate the results obtained by CIBERSORT, another algorithm xCell [19] was performed for the quantification of overall immune cell infiltration and xCell abundance scores of those immune cell types which were significantly different between OA tissues and normal tissues in CIBERSORT results. Expression data from different anatomical structures of knee in osteoarthritis was concatenated in different files. xCell ran with the “rnaseq = FALSE” option, and the immune scores and xCell scores were computed. For comparison purposes, only cell types which could be detected by both xCell and CIBERSORT were selected in this validation process.

### 2.6. Statistical Analyses

Datasets from different structures of knee were analyzed separately. Using a limma R package [20] and a sva R package [21], batch normalization was performed for data from different datasets, including datasets with samples of cartilage, meniscus, and synovial tissue. Dataset with samples of subchondral bone was normalized using a limma R package (Figure 1). Details of batch normalization and normalization are available at https://github.com/Au-CZM. Cases with a CIBERSORT *P* value of <0.05 were included in further analysis. Immune cell profile was obtained for each sample, and mean values for each group (normal and OA) were calculated. Unpaired *T*-test was used to evaluate the difference of continuous variables between normal groups and OA groups. Correlations between continuous and categorical variables were evaluated using the Kruskal-Wallis test. Associations between immune cell subsets were tested by the Pearson correlation coefficient. For univariable analyses of the 22 immune cell subsets, adjustment for multiple testing was performed by calculating *q*-values using the Benjamini-Hochberg method. To analyze if distinct classes of immune cell infiltration are present in different groups, we used hierarchical clustering of immune cell proportions by Ward's method. A combination of the elbow method and the Gap statistic was conducted to explore the likely number of distinct clusters in the data.

All analyses were conducted using R version 3.6, excepting that ROC analyses were performed using SPSS 23.0 statistical software (SPSS Inc., Chicago, IL, USA). All statistical tests performed were two-sided, and the *P* values < 0.05 were considered as statistical significance.

## 3. Results

Datasets with samples of 4 anatomical structures of knee joint, including cartilage, synovial tissues, meniscal tissues, and subchondral bone, can be found in GEO. 17 studies were selected in this study, including 3 studies in knee cartilage, 10 studies in knee synovial tissue, 3 studies in knee meniscal tissue, and 1 study in knee subchondral bone (Table 1) [22–31]. By integrated analysis, 12945 genes for cartilage, 3238 genes for synovial tissue, 6598 genes for meniscal tissue, and 9570 genes for subchondral bone were obtained. Using CIBERSORT algorithm, we first investigated the difference of immune infiltration between normal and OA synovial tissue in 22 subpopulations of immune cells.

### 3.1. Synovial Tissue

**Figure 2:**
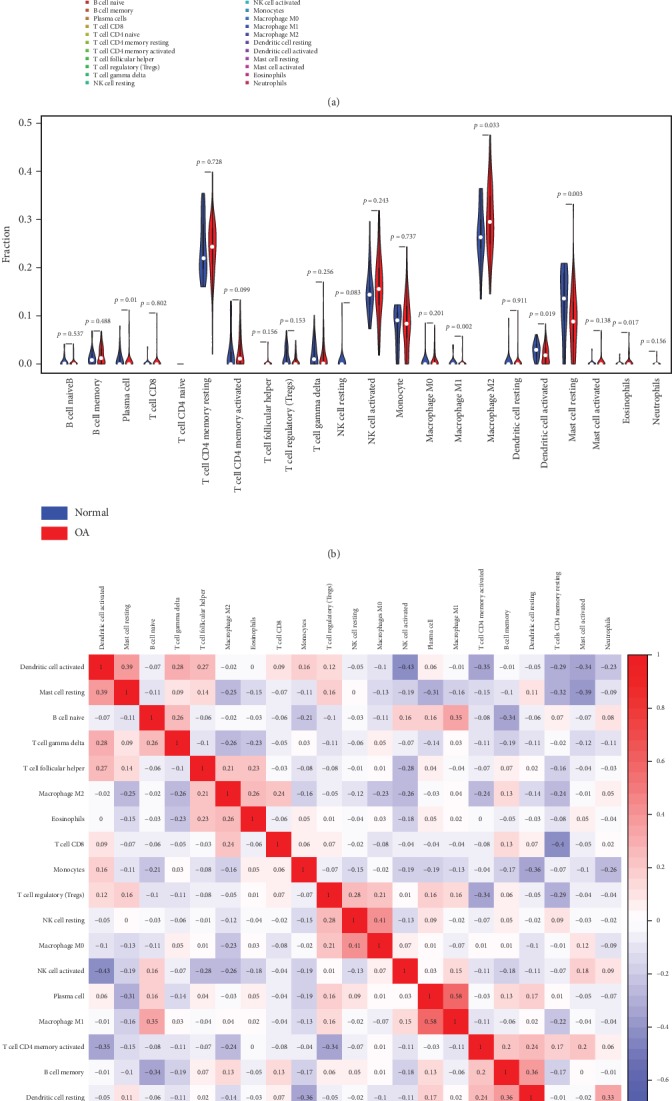
The landscape of immune infiltration in osteoarthritis in synovial tissue. (a) The composition of immune cells for each sample. Total: the average composition of immune cells. **(**b) The difference of immune infiltration between osteoarthritic tissue and normal tissue. (c) Correlation matrix of all 22 immune cell proportions. (d) Heat map of the 22 immune cell proportions. OA: osteoarthritis.

In synovial tissue of knee joint, Figure 2(a) summarized the results obtained from 122 samples with a CIBERSORT *P* value of <0.05. Of these, 30 samples were normal and 92 samples were osteoarthritic.

Overall, in osteoarthritic synovial samples, the most abundant immune cells were M2 macrophages with 30.10%, resting T cell CD4 memory with 23.88%, and activated NK cells with 16.20%, while in normal synovial samples, the most abundant immune cells were M2 macrophages with 26.76%, resting T cell CD4 memory with 24.06%, and activated NK cells with 15.02%.

The differences in proportions of plasma cells, M1 macrophages, M2 macrophages, activated dendritic cells, resting mast cells, and eosinophils between normal tissues and osteoarthritic tissues were statistically significant (Figure 2(b), *P* < 0.05). Higher proportion for above significantly changed cells existed in osteoarthritic tissues, compared with in normal tissues.

The proportions of different infiltrating immune cell subpopulations were correlated weakly to moderately (Figure 2(c), absolute value of correlation coefficient < 0.80). Correlations of M2 macrophages with other immune cell populations by calculating the Pearson correlation coefficients in Figure 2(c) were all weak (absolute value of correlation coefficient < 0.30).

**Figure 3:**
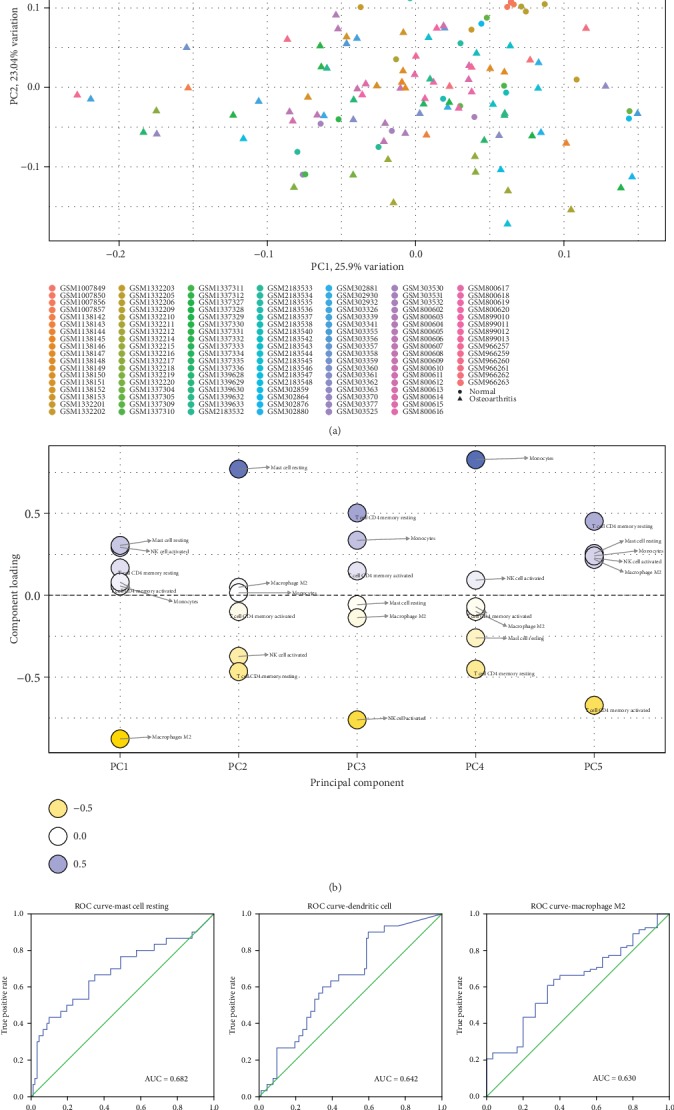
The diagnostic value of composition of infiltrating immune cells for osteoarthritis in synovial tissue. **(**a) Principle component analysis (PCA). (b) Component loading in PCA results. (c) Receiver operating characteristic analysis.

As shown in Figure 2(d), using unsupervised hierarchical clustering based on above-identified cell subpopulation, the samples of pathological and normal could not be clearly separated. PCA was used to assess if the proportions of infiltrating immune cell could be used to differentiate the diagnosis of OA.  Figure 3(a) showed that the diagnosis of OA could not be apparently attributed to the proportions of different infiltrating immune cell subpopulations. The first principle components (PC) accounted for 25.90% variance. M2 macrophages, resting mast cells, and activated NK cells were the major components of PC1, especially M2 macrophages with more than 0.75 component loading (Figure 3(b)).  Figure 3(c) showed that AUC was relatively large in resting mast cells (0.682, (0.560, 0.804)), dendritic cells (0.642, (0.534, 0.750)), and M2 macrophages (0.630, (0.522, 0.738)), in ROC curve analyses. The above-mentioned cells might be related to the pathological mechanism of OA.

Together, these results indicated that aberrant immune infiltration and its heterogeneous in osteoarthritic synovial tissues as a tightly regulated process might have important clinical meanings. It is worth noting that M2 macrophages had a high proportion in the synovial tissue of knee joint, and its proportion in OA patients and normal people had statistical significance. It might have clinical diagnostic significance for OA. In order to develop a diagnostic method using M2 macrophages, the cutoff was determined using the maximum of the Youden index (0.276) based on ROC analysis. The sensitivity and specificity were 0.609 and 0.667, respectively. The diagnostic value of the method only using M2 as diagnostic criteria is limited but really existed.

Therefore, we speculated that M2 macrophages might play an important role in the pathogenesis of OA. However, the composition of immune cells in synovial tissue could not discriminate clearly between normal and OA groups, and other pathogenic factors still needed further investigation.

### 3.2. Subchondral Bone

**Figure 4:**
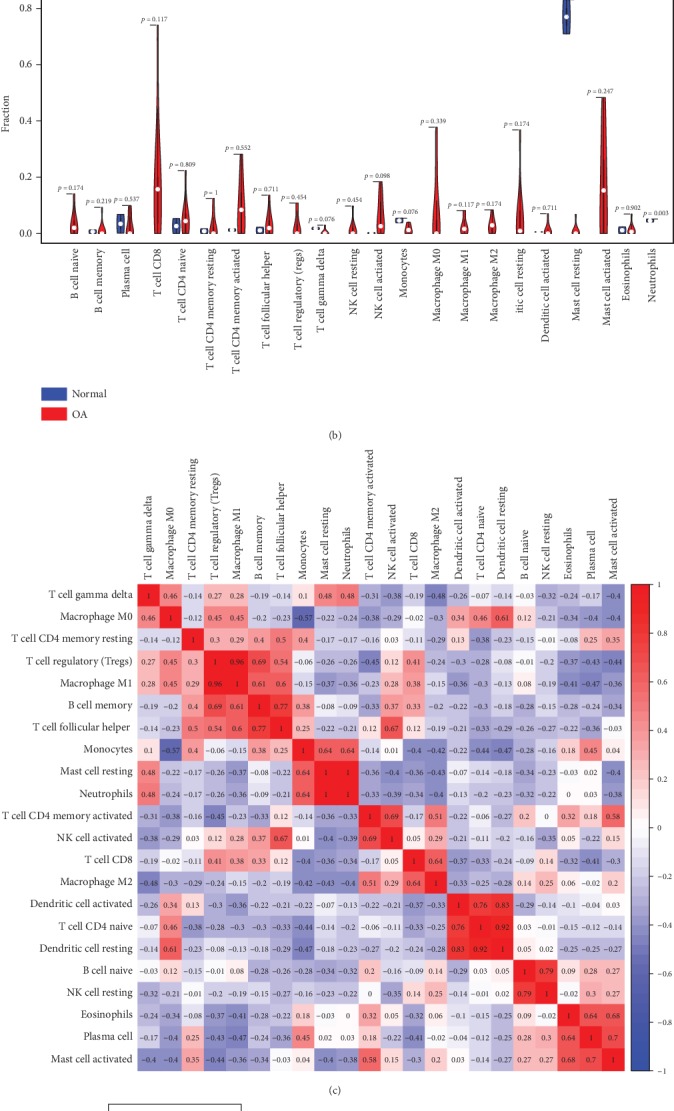
The landscape of immune infiltration in osteoarthritis in subchondral bone. (a) The composition of immune cells for each sample. Normal total: the average composition of immune cells in normal tissue; OA total: the average composition of immune cells in osteoarthritic tissue. (b) The difference of immune infiltration between osteoarthritic tissue and normal tissue. (c) Correlation matrix of all 22 immune cell proportions. (d) Heat map of the 22 immune cell proportions. OA: osteoarthritis.

In subchondral bones of knee joint, Figure 4(a) summarized the results obtained from 11 samples with a CIBERSORT *P* value of <0.05. Of these, 2 samples (GSM1248762, GSM1248767) were normal and other 9 samples were osteoarthritic.

Overall, in osteoarthritic subchondral bones, the most abundant infiltrating immune cells were T cell CD8 with 18.84%, activated mast cells with 17.37%, and activated T cell CD4 memory with 9.12%, while in normal synovial samples, the most abundant infiltrating immune cells were resting mast cells with 76.97%, monocytes with 4.63%, and neutrophils with 4.61%.

The differences in proportions of resting master cells and neutrophils between normal tissues and osteoarthritic tissues were statistically significant (Figure 4(b), *P* < 0.05). Lower proportion for above significantly changed cells existed in osteoarthritic tissues, compared with normal tissues. Considering that 2 normal cases were low number samples, we used all 10 normal cases including low overall infiltration of immune cells and found that the differences in proportions of resting master cells and neutrophils between normal tissues and osteoarthritic tissues were still statistically significant with *P* = 0.005 and *P* < 0.001, respectively.

Correlations between proportions of resting dendritic cells and activated dendritic cells, resting dendritic cells and T cell CD4 naive, M1 macrophages and T cell regulatory, and M2 macrophages and neutrophils were strong (absolute value of correlation coefficient > 0.80, Figure 4(c)). There might be a potential interaction between them.

To further elucidate the role of mast cells in the OA immune cell network, correlations of resting and activated mast cells with other immune cell populations by calculating the Pearson correlation coefficients in Figure 4(c) attracted our attention. Activated mast cells correlated positively with resting T cell CD4 memory, activated T cell CD4 memory, plasma cells, and eosinophils. However, they correlated negatively with T cell gamma delta, M0 and M1 macrophages, T cell regulatory, B cell memory, resting mast cells, neutrophils, and T cell CD8. Resting mast cells correlated positively with T cell gamma delta, monocytes, and neutrophils, while they correlated negatively with M1 macrophages, activated T cell CD4 memory, activated NK cells, T cell CD8, M2 macrophages, B cell naive, and activated master cells. Among them, statistical tests of correlation analysis were significant between activated mast cells and plasma cells, activated mast cells and eosinophils, resting mast cells and monocytes, and resting mast cells and neutrophils.

As shown in Figure 4(d), using unsupervised hierarchical clustering based on the above-identified cell subpopulation in subchondral bone, the samples of pathological and normal could be clearly separated into two discrete groups.

**Figure 5:**
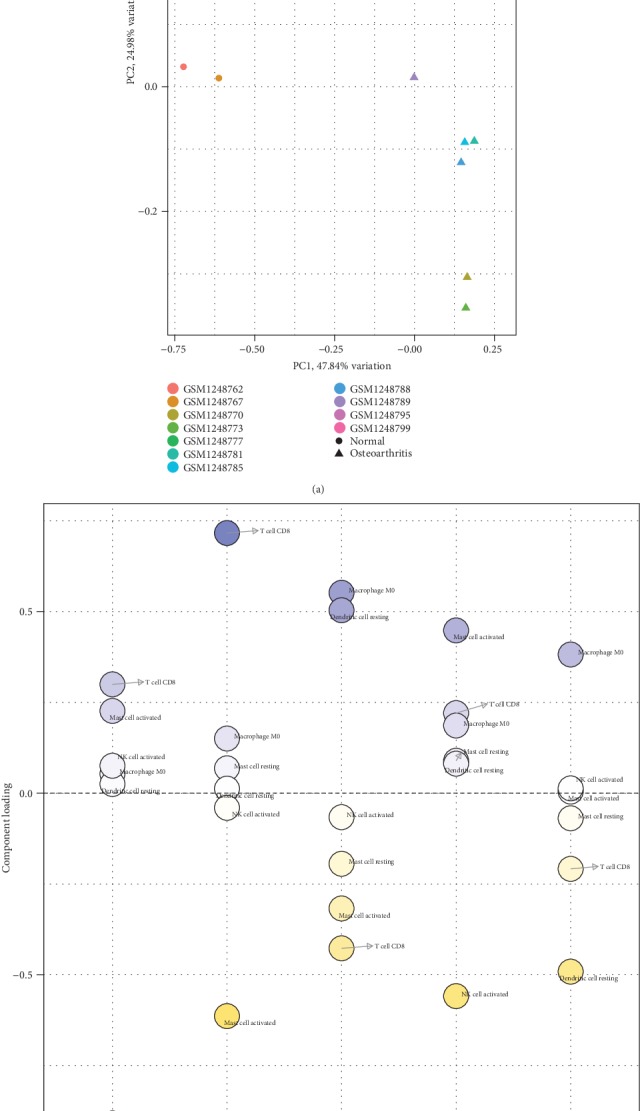
The diagnostic value of composition of infiltrating immune cells for osteoarthritis in subchondral bone. (a) Principle components analysis (PCA). (b) Component loading in PCA results.

In subchondral bones, the proportions of immune cells, from the PCA, displayed distinct group-bias clustering (Figure 5(a)). PC1 appeared to discriminate further between normal and OA groups, with 47.84% variation. Resting mast cells and T cell CD8 were the major component of PC1 (Figure 5(b)). The above-mentioned cells might be related to the pathological mechanism of OA.

Collectively, in subchondral bones, these results indicated that aberrant immune infiltration and its heterogeneous in OA also might have important clinical meanings. It is worth noting that the proportion of master cells was high in knee subchondral bones. And the difference of their proportion in OA patients and normal people had statistical significance. It also had certain clinical diagnostic significance for OA. Therefore, we speculated that master cells played an important role in the pathogenesis of OA. Compared with synovial tissue, the composition of immune cells appeared to discriminate further between normal and OA groups. Thus, the bone immune response in subchondral bones was relatively important pathogenesis.

### 3.3. Meniscus and Cartilage

**Figure 6:**
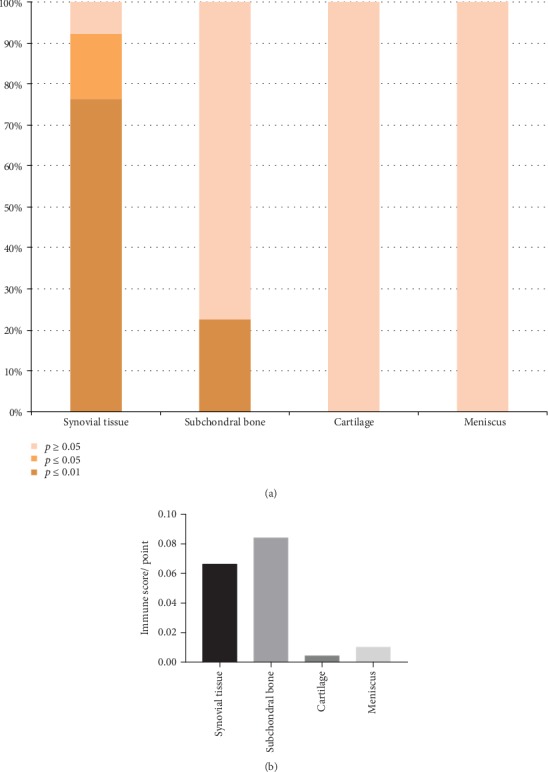
Overall infiltration of immune cells in different osteoarthritis tissues. (a) Overall proportion of immune cells in different osteoarthritis tissues. *P* ≤ 0.01: high infiltration of immune cells; 0.01 < *P* ≤ 0.05: medium infiltration of immune cells; *P* > 0.05: low infiltration of immune cells. (b) Immune scores in different osteoarthritis tissues calculated by xCell.

Using the CIBERSORT algorithm, we found that the CIBERSORT *P* values of 22 subpopulations of infiltrating immune cells in 71 cartilage OA samples and 25 OA meniscus samples from 6 studies were higher than 0.05 (Figure 6(a)).

As mentioned before, the *P* value derived by CIBERSORT could reflect the proportion of a sample that comprises immune cells versus nonimmune cells. This indicated that there was almost no infiltration of immune cells in the meniscus and cartilage of the knee joint. Therefore, we speculated that in the pathogenesis of OA, cartilage and meniscus lesions mainly came from mechanical injury, humoral immunity, and other factors. Figure 6(a) also showed a high degree of immune cell infiltration in synovial OA tissues (76.47%, CIBERSORT *P* ≤ 0.01).

The degree of immune cell infiltration into the tissue is a crucial prognostic factor. To characterize the correlations between immune cell composition and the degree of immune cell infiltration in OA, Pearson correlations of 22 immune cell types with CIBERSORT *P* values were calculated. Finally, we found that activated mast cells were mainly associated with high immune cell infiltration in OA, no matter whether in synovium (correlation coefficient = 0.713) or subchondral bone (correlation coefficient = 0.359). *P* values < 0.002 were considered as statistical significance (Bonferroni correction).

### 3.4. Similar Results Calculated by xCell and CIBERSORT Algorithms

Immune scores obtained by xCell showed immune cell infiltration in the cartilage, and the meniscus was very low (Figure 6(b)), which was similar with the result obtained by CIBERSORT. Moreover, xCell scores of immune cells were mostly consistent with proportions of immune cells calculated by CIBERSORT (Table 2).

## 4. Discussion

In the present study, we applied CIBERSORT to assess differential immune cell infiltration in osteoarthritic tissues and normal tissues in different structures of knee in osteoarthritis.

We found that M2 macrophages infiltrated in the synovium accounted for a high proportion, so the synovium might be as the immunogenic location for M2 macrophages playing an important role in OA. Previous studies have found that there was a certain correlation between macrophages and OA [32, 33]. Takano et al. found that interleukin- (IL-) 1*β* induced by macrophages in the synovium could upregulate calcitonin receptor in a mouse OA model, and calcitonin gene-related peptide was involved in the occurrence of arthritis-related pain [34]. Daghestani et al. have assessed the inflammatory phenotypes predicted by macrophage biomarkers in synovial fluid and blood of patients with KOA. And they found that CD14, CD163 in synovial fluid, and CD163 in serum were associated with a large number of active macrophages, while CD163 and CD14 were associated with formation of osteophyte in knee joint. CD14 was also associated with the severity of knee joint space narrowing, while CD14 in synovial fluid and serum was associated with knee pain [35]. There are different types of macrophages, and Mills et al. proposed the M1–M2 terminology in 2000 [36]. Macrophages activated through a pathway opposite to the classical pathway are referred to as M2 or the alternative pathway. It has been demonstrated that stimuli such as CSF-1, IL-4, IL-10, TGF-*β*, IL-13, fungi, and helminth infections favor M2 subpopulation polarization, delivering IL-10 in high concentrations, and IL-12 in low amounts. A series of studies have found that immune-suppressive, proangiogenic M2 macrophages play a central role in responses to parasites, tissue remodeling, angiogenesis, and allergic diseases [37, 38]. In addition, CD163 is one of the markers of macrophage M2 [39]. Combined with our result that M2 macrophages were correlated to OA, we speculated that infiltrating M2 macrophages in synovium might play a vital role in the pathogenesis of OA.

Subchondral bone is another important component in the progress of OA. Osteosclerosis in subchondral bone caused by abnormal changes of subchondral bone could occur in the early stage of OA. Moreover, some studies have found that subchondral bone might be the initial cause of osteoarthritis [40, 41]. Therefore, current research on the pathogenesis of osteoarthritis and the research about new treatment of KOA have focused on the role of subchondral bone in the pathogenesis and progression of osteoarthritis [42]. Most of previous studies related to subchondral bone and the pathogenesis of osteoarthritis focused on bone metabolism and biomechanical mechanisms [43–45]. Mast cells are the most important effector cells in the innate immune system. They are transformed from hematopoietic cells produced by the precursors of pluripotent bone marrow stem cells. Mast cells have attracted much attention in the field of rheumatoid arthritis [46–48]. Ruschpler et al. have found that mast cells played an important role in the pathogenesis of rheumatoid arthritis [49]. However, compared with studies in rheumatoid arthritis, the number of studies about mast cells in osteoarthritis is far less. In the present study, less resting mast cells and more activated mast cells were found in the subchondral bone of OA patients. Therefore, the immunological study of subchondral bone in osteoarthritis and the influence of mast cells on it should be paid more attention.

Moreover, we found activated mast cells were mainly associated with high immune cell infiltration in OA, in both of synovium and subchondral bone. Mast cells could be activated by different stimuli [50]. However, CIBERSORT enumerates specifically IgE-activated mast cells because the gene expression signature used for deconvolution was obtained from mast cells stimulated by IgE [15]. Mast cells are key regulators of immune effector cells [51]. Therefore, their activation could be a desired aim of immunotherapy. Moreover, we revealed the potential interaction between these immune cells and other immune cells in our study.

The present study only focused on immune cell infiltration. However, other forms of immune response remain nonnegligible. In the degeneration of articular cartilage, extracellular matrix, which protects special surface antigens on chondrocyte from the immune system, disappeared and immune barrier was destroyed. Huber-Lang et al. found that on the surface of posttraumatic debris, a variety of products activated by complements were found on the surface of chondrocyte [52]. Jong et al. have proved that cartilage proteoglycan peptide located in G1 domain could induce T cell reaction and promote cartilage degradation in patients with OA. Moreover, in proteoglycan and Yersinia outer proteins, there is a same amino acid region, 263-283 sites, which could induce immune response in patients with OA [53]. Frisenda et al. used heterogeneous type II collagen to immunize mice, which could induce arthritis, suggesting that cartilage collagen was also a potential target of autoimmune reaction [54]. Combined with the results of our study, it could be speculated that in the cartilage of patients with OA, the immune response might occur more in the form of humoral immunity or other ways that did not require the infiltration of immune cells.

Similarly, type I collagen is one of the components in meniscus. When the meniscus was damaged, the exposed type I collagen could also stimulate autoimmune response [55]. In addition, inflammatory mediators have been shown to be able to cooperate with nitric oxide, inhibit the synthesis of collagen II and proteoglycan, and accelerate their degradation [56]. Combining with the relative lack of blood supply and lymph nodes in the meniscus and cartilage, the results of the present study excluded the influence of immune cell infiltration in the meniscus and cartilage on OA to a certain extent.

xCell is a novel gene signature-based method for inferring 64 cell types including stromal cells and stem cells. Therefore, xCell needs more genes for analysis and calculation than CIBERSORT. In the present study, GSE12021 for synovial tissue was not selected for xCell analysis due to the insufficient number of genes.

There are still some limitations to be acknowledged. First, healthy knee samples were rare, and normal samples in our study were mostly collected after amputation or osteotomy which potentially influences immune infiltration. Second, in order to enlarge our sample size, several studies from different platform were combined. Although we conducted statistical methods to eliminate the bias, heterogeneity in these data still impeded the repeatability to some extent. Third, data in the present study only could provide the correlation analysis between OA and immune cells, instead of the exploration of the cause and effect relationship. However, as mentioned above, previous studies have demonstrated that immunological mechanisms play the vital role in the pathogenesis of OA. Thus, we speculated that different infiltrating immune cell subpopulations were the potential reason of OA. Finally, the present study was based on publicly accessible array datasets. Some basic characteristics of patients including age were missing. Meanwhile, the immunologic function could be influenced by many factors, including age and gender [6]. However, pathological changing is always considered as the main factor of the local infiltration of immune cells. Therefore, sometimes other factors were neglected when analyzed [18, 57, 58]. Our study mainly proposed some new ideas in immunology for researches related to knee osteoarthritis. Further researches are still necessary to validate our speculation.

## 5. Conclusion

The immune cell composition in OA differed substantially from that of healthy joint tissue, while it also differed in different anatomical structures of the knee. Meanwhile, activated mast cells were mainly associated with high immune cell infiltration in OA. Furthermore, we speculate M2 macrophages in synovium and mast cells in subchondral bone may play an important role in the pathogenesis of OA.

## Figures and Tables

**Table 1:** Profiling datasets of Gene Expression Omnibus (GEO).

Tissue source	GEO ID	Normal	Case	Platform	Year^∗^	Country	Author^∗∗^
Knee cartilage	GSE43191	0	23	GPL11532 [HuGene-1_1-st] Affymetrix Human Gene 1.1 ST Array	2016	Spain	Fernández-Tajes J
	GSE64394	5	2	GPL6244 [HuGene-1_0-st] Affymetrix Human Gene 1.0 ST Array	2018	USA	Bhutani N
	GSE98460	0	46	GPL16686 [HuGene-2_0-st] Affymetrix Human Gene 2.0 ST Array	2019	USA	Muhammad Farooq Rai

Knee synovial tissue	GSE12021	13	20	GPL96 [HG-U133A] Affymetrix Human Genome U133A Array GPL97 [HG-U133B] Affymetrix Human Genome U133B Array	2018	Germany	Huber R
	GSE32317	0	19	GPL570 [HG-U133_Plus_2] Affymetrix Human Genome U133 Plus 2.0 Array	2019	USA	Scanzello CR
	GSE36700	0	5	GPL570 [HG-U133_Plus_2] Affymetrix Human Genome U133 Plus 2.0 Array	2019	Belgium	Lauwerys BR
	GSE39340	0	7	GPL10558 Illumina HumanHT-12 V4.0 Expression BeadChip	2018	China	Xiaotian C
	GSE41038	4	3	GPL6883 Illumina HumanRef-8 v3.0 Expression BeadChip	2019	Australia	Thomas GP
	GSE46750	0	12	GPL10558 Illumina HumanHT-12 V4.0 Expression BeadChip	2018	Belgium	Lambert C
	GSE55235	10	10	GPL96 [HG-U133A] Affymetrix Human Genome U133A Array	2018	Germany	Thomas H
	GSE55457	10	10	GPL96 [HG-U133A] Affymetrix Human Genome U133A Array	2018	Germany	Kinne RW
	GSE55584	0	6	GPL96 [HG-U133A] Affymetrix Human Genome U133A Array	2018	Germany	Peter S
	GSE82107	7	10	GPL570 [HG-U133_Plus_2] Affymetrix Human Genome U133 Plus 2.0 Array	2019	Netherlands	de Vries M

Knee meniscus	GSE19060	3	5	GPL570 [HG-U133_Plus_2] Affymetrix Human Genome U133 Plus 2.0 Array	2019	USA	Sun Y
	GSE52042	0	8	GPL17882 Microarrays Inc. Human MI Ready Array–49K Genomic Array	2014	Germany	Von der Heyde S
	GSE98918	12	12	GPL20844 Agilent-072363 SurePrint G3 Human GE v3 8x60K Microarray	2018	USA	Zhang B

Knee subchondral bone	GSE51588	10	40	GPL13497 Agilent-026652 Whole Human Genome Microarray	2018	USA	Chou CH

^∗^Year: last update date. ^∗∗^Author: contact name.

**Table 2:** Comparison of immune cell abundance in osteoarthritis subchondral bones and osteoarthritis synovial tissues calculated by xCell and CIBERSORT algorithms.

	Cell type	xCell score	Correlation between xCell and CIBERSORT		Qualitative consistency xCell vs. CIBERSORT
			Correlation coefficient	*P* value	
Subchondral bone	Neutrophils	0.0039 ± 0.00175	0.602	<0.001	Yes

Synovial tissue	Plasma cells	0.0066 ± 0.00163	0.555	<0.001	Yes
	Macrophage M1	0.0067 ± 0.00101	-0.087	0.410	No
	Macrophage M2	0.0071 ± 0.00120	0.233	0.026	Yes
	Eosinophils	0.0001 ± 0.00006	0.075	0.479	Yes^∗^

^∗^Qualitative consistency without statistical significance.

## Data Availability

The data used to support the findings of this study are available from the corresponding author upon request.
